# Key Vital Signs Monitor Based on MIMO Radar

**DOI:** 10.3390/s25134081

**Published:** 2025-06-30

**Authors:** Michael Gottinger, Nicola Notari, Samuel Dutler, Samuel Kranz, Robin Vetsch, Tindaro Pittorino, Christoph Würsch, Guido Piai

**Affiliations:** 1 Institute for Electronics, Sensorics and Actorics (ESA), Ostschweizer Fachhochschule, 9470 Buchs, Switzerland; samuel.kranz@ost.ch (S.K.); tindaro.pittorino@ost.ch (T.P.); guido.piai@ost.ch (G.P.); 2 Institute for Computational Engineering (ICE), Ostschweizer Fachhochschule, 9470 Buchs, Switzerland; nicola.notari@ost.ch (N.N.); samuel.dutler@ost.ch (S.D.); robin.vetsch@ost.ch (R.V.); christoph.wuersch@ost.ch (C.W.)

**Keywords:** computer-aided diagnosis, convolutional neural networks, displacement measurements, machine learning, MIMO radar, patient monitoring, sleep apnea

## Abstract

State-of-the-art radar systems for the contactless monitoring of vital signs and respiratory diseases are typically based on single-channel continuous wave (CW) technology. This technique allows precise measurements of respiration patterns, periods of movement, and heart rate. Major practical problems arise as CW systems suffer from signal cancellation due to destructive interference, limited overall functionality, and a possibility of low signal quality over longer periods. This work introduces a sophisticated multiple-input multiple-output (MIMO) solution that captures a radar image to estimate the sleep pose and position of a person (first step) and determine key vital parameters (second step). The first step is enabled by processing radar data with a forked convolutional neural network, which is trained with reference data captured by a time-of-flight depth camera. Key vital parameters that can be measured in the second step are respiration rate, asynchronous respiratory movement of chest and abdomen and limb movements. The developed algorithms were tested through experiments. The achieved mean absolute error (MAE) for the locations of the xiphoid and navel was less than 5 cm and the categorical accuracy of pose classification and limb movement detection was better than 90% and 98.6%, respectively. The MAE of the breathing rate was measured between 0.06 and 0.8 cycles per minute.

## 1. Introduction

Sleep apnea leads to repeated pauses in breathing during sleep, resulting in reduced oxygen supply to the body and increased risk of cardiovascular disease [1] and cognitive impairment, and may be an important risk factor for stroke [2] or sudden unexpected death in infancy [3]. Polysomnography is the gold standard for diagnosing sleep apnea and requires many different sensors, including electroencephalography (EEG), electrooculography (EOG), electromyography (EMG), and electrocardiography (ECG) sensors, to measure respiration and oxygen levels [4]. Due to the conditions in a sleep laboratory and the numerous wired sensors attached to the body, patients can have problems falling asleep, and the manual evaluation of the data by experts is cumbersome and expensive.

To improve sleep comfort and allow for a diagnosis at home, alternative devices are available [5], for instance, wrist oximeters or accelerometers [6], respiratory effort belts, micro-electro-mechanical systems (MEMS) for measuring nasal air flow [7], and continuous positive airway pressure devices (CPAPs) [8]. Contactless remote monitoring based on microwave radar completely avoids any influence on the quality of sleep [9] and allows for the automated evaluation of data for the detection of apnea events. Unlike with video [10] or acoustic recordings [11], the privacy of patients is preserved when using radar. To this end, sensors can be placed above the bed, on the bedside, or below the mattress [12]. Numerous publications explain the operation of single-channel continuous wave (CW) radar systems for vital parameter detection [13], such as breathing rate, breathing interruptions, and heart rate [14]. When monitoring a person in a bed, a CW radar receives a superposition of signals from all reflection points, since the observed body surface is curved and not comparable to a flat plate [15]. Therefore, a CW radar is unable to distinguish individual regions of the body and the measurement does not provide distance or angular information, so the position of the person cannot be reliably measured and the functionality of the remote monitoring system is limited. In addition, destructive interference can occur when multiple reflecting objects or human beings are in the field of view (FoV).

Modulated radio-frequency (RF) signals, for example, those in the form of an ultra-wideband (UWB) impulse radio [16] or frequency-modulated continuous wave (FMCW) [17], ease the situation by providing distance information, increasing the detection reliability, and enhancing the suppression of surrounding clutter [18]. To further expand the amount of information by adding angular data, multichannel radar with antenna arrays either on the receive (RX) or transmit (TX) side, or a combination of both variants in a multiple-input multiple-output (MIMO) system, can be employed [19]. On the one hand, MIMO radar systems with few TX and RX channels allow for the simultaneous measurement of the locations and vital parameters of multiple individuals [20]. Due to the low angular resolution in the far-field, each test subject is visible as a point-like target, and individual parts of their body cannot be identified. On the other hand, MIMO imaging systems with a large number of channels and wide RF bandwidth are known from security applications, for instance, at airports [21]. Radar images captured with a lateral resolution of ≈2 mm reveal small details. Because of their large dimensions, high power consumption, significant privacy concerns, and high costs, even the more compact personnel imaging systems [22] are not a viable solution for monitoring vital signs at home. A combination of camera images and radar images for this purpose is described in, for example, [23]. In this application, the camera was used not only for training but also during measurement.

To enable a reliable diagnosis of sleep apnea, the measurement of related physiological parameters with Doppler radar can provide relevant information to increase confidence [24]. For instance, events of paradoxical breathing can be recognized with two separate near-field coupled CW sensors on the body [25] or by two sensors with directive antennas focused on the chest and abdomen region [26]. It was shown in [27] that variations in the photoplethysmogram (PPG) are also related to sleep apnea and that screening can be performed by an analysis of heart rate variability (HRV). The radar-based estimation of HRV is feasible with the patient positioned below a radar pointing at the chest region [28]. An association with periodic limb movements has been shown in 24–48% of patients with obstructive sleep apnea [29]. During the night, a person in a bed is not in a static position or orientation, which is disadvantageous for all these approaches, preventing their direct application to apnea screening at home. To overcome the lack of position information, a convolutional neural network (CNN) approach using WiFi radio data in the form of heatmaps is described in [29]. This method was implemented by supervised training with RGB reference images.

**Figure 1:**
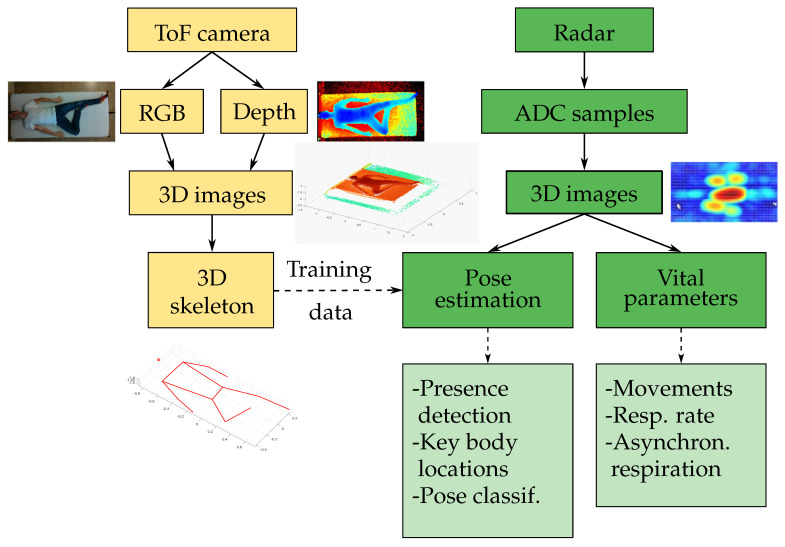
Overview of the main operations and functions with ToF camera data (yellow) and radar data (green).

The aim of the present study was to build and evaluate a radar-based measurement system for reliable vital signs monitoring during sleep at home, with a particular focus on the localization of the measured person on the bed and the recognition of periods with asynchronous respiratory movement of the chest and abdomen (often observed during paradoxical breathing episodes [30]). We propose a compact 8 cm × 8 cm MIMO radar positioned next to the bed at a distance of approximately 1 m to the person in conjunction with a CNN model for localization. In contrast to state-of-the-art single-channel systems, the MIMO radar is capable of providing a 3D point cloud that allows for the estimation of the position, sleep pose, and distinction of different parts of the body. This is a compromise that minimizes complexity while achieving a sufficiently high resolution. An overview of the system setup with the important features of our solution is depicted in Figure 1. The 3D joint coordinates of a person are extracted using Google MediaPipe Pose [31] from RGB and depth images captured by a Microsoft Azure Kinect DK time-of-flight (ToF) camera (left branch). This information only serves as training data for the pose estimation via the CNN, comparable to [32,33]. The right branch shows the processing of the radar data recorded with a 60 GHz Vayyar IMAGEVK-74 [34] MIMO system with 20 TX and 20 RX channels and 5.2 GHz RF bandwidth. After preprocessing, the key locations of the body—the chest, abdomen, and head—are determined from 3D radar images, and periods with movements are identified. The system then characterizes the respiration rate and identifies limb movements and asynchronous respiratory movements of chest and abdomen.

The remainder of this article is structured as follows: In Section 2, the radar image reconstruction is explained briefly, followed by a description of the data preprocessing. A CNN approach for pose estimation and algorithms for the detection of body movements and breathing rate and the recognition of periods with asynchronous respiratory movement of the chest and abdomen are proposed in Section 3. The measurement setup and results of the measurement campaign are presented in Section 4. Practical tests with 23 volunteers are described to compare the accuracy of the radar-based estimation of the pose and vital parameters to reference data recorded with a ToF camera and inductive plethysmography belts. Finally, a summary of the results and an outlook are given in Section 5.

## 2. Theory and Preprocessing

In the first part of this section, image reconstruction for an L-shaped MIMO array is presented briefly, starting from the general radar imaging model. In the second part, the preprocessing steps to create input data for the algorithms in Section 3 are given.

### 2.1. MIMO Radar Signal Model and Image Reconstruction

Assuming an arbitrary time-varying target scene in the near-field of the aperture, defined by a reflection coefficient $\sigma \left(r,t\right)$ and isotropically radiating antenna elements, the received signal was

(1) $$\begin{array}{c} s\left(t,f,x_{t},z_{t},x_{r},z_{r}\right)=\frac{1}{16\pi^{2}}\underset{V}{\;\int \int \int \;}\sigma \left(r,t\right)\cdot \frac{e^{-jk\left(f\right)\left(R_{t}+R_{r}\right)}}{R_{t}R_{r}}d^{3}r, \end{array}$$

where $x_{t/r}$ and $z_{t/r}$ denote the *x*- and *z*-coordinates of the transmit and receive elements, respectively [35]. All antennas were located in the y=0 plane, with $c_{0}$ denoting the phase velocity. The variables

(2) $$\begin{array}{c} R_{t/r}=\sqrt{{\left(x_{t/r}-x\right)}^{2}+y^{2}+{\left(z_{t/r}-z\right)}^{2}} \end{array}$$

represented the Euclidean distances from the transmit and receive elements to a point r in space, and $k\left(f\right)=2\pi f/c_{0}$ was the wave number as a function of the frequency *f*. Hence, the signal in (1) was the coherent superposition of all scattered fields in the volume V, and image reconstruction could be performed directly using a spatially matched filter [36] that compensated for the phase induced by the propagation along each path.

**Figure 2:**
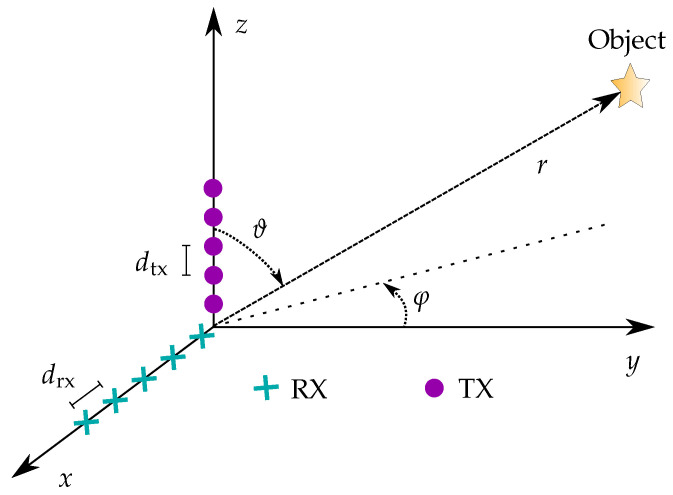
Coordinate system with MIMO radar based on an L-shaped antenna array comprised of RX and TX elements (cyan and purple, respectively).

Due to the high computational load, direct reconstruction is often not the preferred technique. To simplify the signal model and the corresponding imaging method, the L-shaped antenna array used in this application was considered (see Figure 2). In this case, the TX elements were uniformly distributed on the *z*-axis at an inter-element distance of $d_{\mathrm{tx}}$, and the RX elements were located on the *x*-axis with a spatial separation of $d_{\mathrm{rx}}$. All scatterers were assumed to be in the far-field of the antenna array. By introducing a spherical coordinate system comparable to [37], where the locations of scatterers depended on the distance to the origin *r*, azimuth angle φ, and elevation angle ϑ, the received signals were

(3) $$\begin{array}{cc} \; & s\left(t,\ell ,m,n,r,\varphi ,\vartheta \right)\approx \\ \; & \;\;\;\frac{1}{16\pi^{2}}\underset{A}{\;\int \int \;}\sigma \left(r,t\right)\cdot \frac{e^{-j2k_{0}r}}{r^{2}}\cdot e^{-j\frac{4\pi }{c_{0}}\left(\ell -\frac{L}{2}\right)\Delta fr}\cdot e^{-jk_{0}\left[\left(m-1\right)d_{\mathrm{rx}}\cos\vartheta \sin\varphi +\left(n-1\right)d_{\mathrm{tx}}\sin\vartheta \right]}d\nu \left(t\right). \end{array}$$

The variables m∈{1,…,M} and n∈{1,…,N} denoted the number of RX and TX elements, respectively, with the arrays consisting of *M* RX and *N* TX antennas in total. It was further assumed that a reflection of electromagnetic waves only occurred at the skin of a person as the penetration depth was very low, for example, 0.43 mm for a frequency of 60 GHz [38]. Hence, the volume integral could be replaced by a scalar surface integral over A with an infinitesimal element $d\nu \left(t\right)$ of this surface. Moreover, the modulation was chosen as a stepped-frequency continuous wave (SFCW), defined by ℓ∈{1,…,L} uniformly distributed steps of Δf. The instantaneous frequency in (3) became

(4) $$\begin{array}{c} f=f_{0}+\left(\ell -L/2\right)\Delta f \end{array}$$

and the wave number $k_{0}=2\pi f_{0}/c_{0}$ corresponded to the carrier center frequency $f_{0}$. For the desired application, SFCW was appealing due to its simplicity and the low hardware requirements on, for instance, the generation of this waveform and the sampling rate of the analog-to-digital converter (ADC).

Other waveforms can be used readily, for example, FMCW or orthogonal frequency division multiplexing, which are popular modulation formats that are typical in applications such as automotive radars [39]. For the MIMO operation, individual TX elements were activated via time division multiplexing (TDM) to ensure orthogonality. Due to the very slow movements in this application, there were no further measures required to compensate for the phase shift caused by the sequential activation of TX channels. If the induced phase changes were too high, a motion compensation according to [40] could be used.

Using a window function $w\left[\cdot \right]$ to suppress the level of sidelobes along all dimensions, the spatially matched filter for image reconstruction could be simplified to

(5) $$\begin{array}{cc} I\left(t,r,\varphi ,\vartheta \right) & =\frac{1}{LMN}\sum_{n=1}^{N}\sum_{m=1}^{M}\sum_{\ell =1}^{L}\{w\left[\ell ,m,n\right]\cdot s\left(t,\ell ,m,n,r,\varphi ,\vartheta \right)\cdot e^{j\frac{4\pi }{c_{0}}\left(\ell -\frac{L}{2}\right)\Delta fr}\\ \; & \;\;\;\cdot e^{jk_{0}\left[\left(m-1\right)d_{\mathrm{rx}}\cos\vartheta \sin\varphi +\left(n-1\right)d_{\mathrm{tx}}\sin\vartheta \right]}\}. \end{array}$$

The summation could be replaced by a three-dimensional fast Fourier transform (FFT) to drastically reduce the required computation time [37]. To estimate the vital parameters, such an image was created for each discrete time step, especially to assess changes of the phase.

### 2.2. Preprocessing

For each discrete time step $t_{i}$, after applying a three-dimensional FFT with a Hanning window function to the MIMO radar data, a complex array $I\left[t_{i},r_{j},\varphi_{k},\vartheta_{l}\right]$ with dimensions $N_{r}\times N_{\varphi }\times N_{\vartheta }$ was obtained, where $N_{r}$, $N_{\varphi }$, and $N_{\vartheta }$ represent the number of FFT points considered in the azimuthal, elevation, and distance dimensions lying close to the bed region. To limit the volume of data stored, the following additional steps were applied:

- Recorded data were divided into frames with time windows of τ=25s duration with a Δτ=5s overlap. The window length was chosen to allow a reliable breathing rate estimation by including several breathing periods (typically four to six) in one window. The overlap allowed a smooth transition of the estimated skin movement between consecutive windows.
- For each window, the mean amplitude $\bar{A}\left[r_{j},\varphi_{k},\vartheta_{l}\right]={\langle \left|I\left[t,r_{j},\varphi_{k},\vartheta_{l}\right]\right|\rangle }_{\tau }$ over time of the radar frames data was calculated. For each combination of azimuth $\varphi_{k}$ and elevation angle $\vartheta_{l}$, the distance index with the largest mean amplitude
  (6) $$\begin{array}{c} j_{\mathrm{MA}}\left[\varphi_{k},\vartheta_{l}\right]=\underset{j}{arg max}\left\{\bar{A}\left[r_{j},\varphi_{k},\vartheta_{l}\right]\right\} \end{array}$$
  was found. The values $I\left[t_{i},r_{j_{MA}},\varphi_{k},\vartheta_{l}\right]$ measured at these distances were stored and used for subsequent evaluations.
- The distances with the largest amplitude
  (7) $$\begin{array}{c} r_{\mathrm{MA}}\left[t_{i},\varphi_{k},\vartheta_{l}\right]=\underset{r_{j}}{arg max}\left\{\left|I\left[t_{i},r_{j},\varphi_{k},\vartheta_{l}\right]\right|\right\} \end{array}$$
  for each azimuth–elevation combination and time step $t_{i}$ were also stored.

The algorithms and procedures described in the following sections are based on this reduced set of preprocessed radar data.

## 3. Parameter Estimation

In this section, the algorithms used to estimate different parameters from MIMO radar data are described. The first part of the estimation consisted of recognizing if a person was present on the bed and estimating the pose and location of key body parts—the head, chest, and abdomen—as well as recognizing the periods during which the person moved (e.g., pose changes or limb movements). Based on this information, breathing-induced movements of the chest and abdomen were tracked separately to quantify the breathing rate and recognize asynchronous chest–abdomen movement.

### 3.1. Pose Estimation

In the first stage, the relevant information regarding the presence and position of a person on the bed was estimated. The procedure consisted of three distinct steps:

1. recognition of the presence of a person sleeping on the bed;
2. classification of the pose of the person sleeping on the bed as supine, prone, or lateral;
3. localization of the key skeleton joints (xiphoid, navel, shoulders, and hips (the other skeleton joints were omitted because they were considered unnecessary for the quantification of vital parameters and much more difficult to detect than the torso due to their smaller size and higher surface curvature.)) of the sleeping person, from which the location of key body parts of interest, i.e., the chest, abdomen, and head, could be estimated.

The algorithms developed to tackle these three tasks took as input a single window of reduced radar data and returned the corresponding output. The first step was shared among the algorithms and consisted of calculating a set of indicators based on the time evolution of the phase and amplitude of the measured reflected radar signal as well as the distance of maximum reflection amplitude at each azimuth–elevation combination $r_{\mathrm{MA}}$. The goal was to reduce the dimensionality of the input data by removing the time dependence and constructing quantities that contained both information about the location and the amplitude of reflections, as well as information about locations where movements with a frequency falling into the typical respiration frequency range were observed.

Five different indicators or features have been constructed:

- *Mean reflection amplitude*: The mean of the reflection amplitude over the time window for each azimuth–elevation pair was
  (8) $$\begin{array}{c} \bar{A}\left[\varphi_{k},\vartheta_{l}\right]={\langle \left|I\left(t,r_{j_{\mathrm{MA}}},\varphi_{k},\vartheta_{l}\right)\right|\rangle }_{\tau }. \end{array}$$
- *Phase evolution indicators*: For each azimuth–elevation pair, the time evolution of the phase was first unwrapped and then the Fourier transform of the unwrapped signal was calculated. The magnitudes of the Fourier transform values were first normalized. The mean and maximum normalized magnitude in the frequency range $\nu_{\mathrm{RR}}\in \left[0.09\;\mathrm{Hz},0.70\;\mathrm{Hz}\right]$ (the expected respiration rate range) were calculated and represented two additional indicators:
  (9) $$\begin{array}{cc} \alpha_{1}\left[\varphi_{k},\vartheta_{l}\right] & =\frac{1}{C\left[\varphi_{k},\vartheta_{l}\right]}{\langle \left|F\{\zeta \left(t,\varphi_{k},\vartheta_{l}\right)\}\right|\rangle }_{\nu_{\mathrm{RR}}},\\ \alpha_{2}\left[\varphi_{k},\vartheta_{l}\right] & =\frac{1}{C\left[\varphi_{k},\vartheta_{l}\right]}\max_{\nu_{\mathrm{RR}}}\left|F\{\zeta \left(t,\varphi_{k},\vartheta_{l}\right)\}\right| \end{array}$$
  with
  (10) $$\begin{array}{cc} \zeta \left(t,\varphi_{k},\vartheta_{l}\right) & =U\{\arg\left\{I\left(t,r_{j_{\mathrm{MA}}},\varphi_{k},\vartheta_{l}\right)\right\}\}\;\mathrm{and}\\ C\left[\varphi_{k},\vartheta_{l}\right] & =\int \left|F\{\zeta \left(t,\varphi_{k},\vartheta_{l}\right)\}\right|d\nu , \end{array}$$
  where F{·} and U{·} denote the Fourier transform (implemented as an FFT) and the phase unwrapping operator, respectively.
- *Distance evolution indicators*: For each azimuth–elevation pair, the standard deviation (std) and the 95 percentile $P_{95}$ of the values of the distance with maximum amplitude $r_{\mathrm{MA}}$ were calculated and represented two additional indicators:
  (11) $$\begin{array}{cc} \beta_{1}\left(\varphi_{k},\vartheta_{l}\right) & =\mathrm{std}\left(r_{\mathrm{MA}}\left(t,\varphi_{k},\vartheta_{l}\right)\right)\;\mathrm{and}\\ \beta_{2}\left(\varphi_{k},\vartheta_{l}\right) & =P_{95}\left(r_{\mathrm{MA}}\left(t,\varphi_{k},\vartheta_{l}\right)\right). \end{array}$$

For all features, the values at the points with a mean reflection amplitude below a fixed threshold ${\bar{A}}_{\mathrm{TS}}$ were neglected and substituted with the minimum value attained at the other points. The goal of this step was to remove noise-dominated resolution cells from the indicator values.

**Figure 3:**
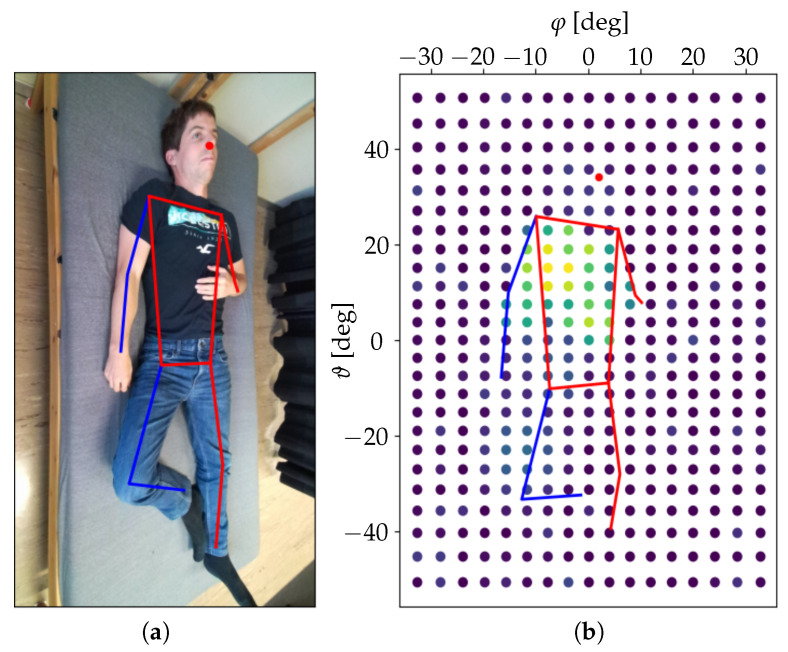
(**a**) RGB image from the Azure Kinect ToF camera used by a skeleton regression algorithm to calculate the reference data for joint positions. (**b**) Example of the values of the second phase evolution indicator $\alpha_{2}\left[\varphi_{k},\vartheta_{l}\right]$ extracted from the radar data in the azimuth–elevation plane with a person measured in supine position. Yellow color corresponds to the maximum.

For each indicator, two 2D arrays of values were created. The first consisted of the indicator values for each azimuth–elevation pair $\left(\varphi_{k},\vartheta_{l}\right)$. The second was built by averaging the indicator values along the azimuthal dimension and thus obtaining one value for each elevation–distance pair $\left(\vartheta_{l},r_{j}\right)$. An example of an azimuth–elevation array obtained for the second phase evolution indicator $\alpha_{2}$ during one of the measurements presented in Section 4 is shown in Figure 3.

To solve the problem of presence recognition, a simple binary classifier was used, which counted the number of azimuth–elevation pairs with a value of the first phase evolution indicator $\alpha_{1}$ above a fixed threshold $\alpha_{1\mathrm{TS}}$ and classified the window as “person present” if this number was above a fixed amount. The intuition behind this approach was that if a person was sleeping on the bed, then within a solid angle around the person’s torso, the radar would measure the relevant displacement caused by the respiration-induced skin movement.

**Figure 4:**
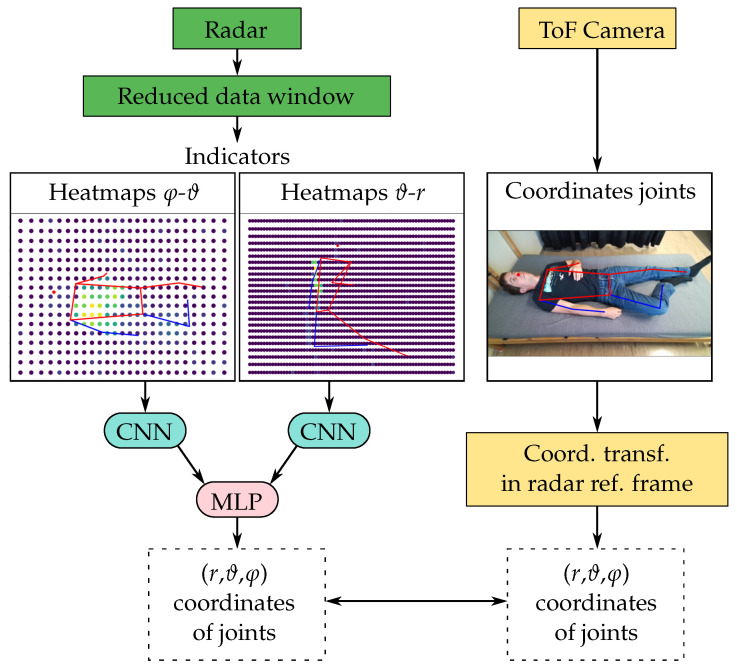
Schematic representation of the pipeline used to build a regression model for the key skeleton joint locations. The skeleton joints plotted on the images represent the positions estimated from the RGB image.

The approach used to tackle the problem of localizing the key skeleton joints of the torso was inspired by the work presented in [32,33] and is summarized in Figure 4. The input of the model was a reduced radar data window and the output was an array containing the azimuthal, elevation, and distance coordinates of the xiphoid, navel, right shoulder, left shoulder, right hip, and left hip of the measured person. The azimuth–elevation and distance–elevation arrays of the five indicators $\bar{A}$, $\alpha_{1}$, $\alpha_{2}$, $\beta_{1}$, and $\beta_{2}$ calculated from the radar data were first centered and normalized and then treated as separate input heatmaps (with five channels each) to a forked CNN architecture. Each of the two CNN forks was built out of three convolutional layers followed by a max-pooling layer and a dropout layer. The data from the two forks were then flattened, concatenated, and sent through a multilayer perceptron (MLP) with four dense layers, two dropout layers, and a final dense layer.

The ground truth data used to train and test the model were constructed based on the positions of the skeleton joints estimated from data collected with a Microsoft Azure Kinect DK ToF camera (RGB image and depth data). The estimation of shoulder and hip joints position was perfomed with Google MediaPipe Pose [31] applied to the RGB image. The obtained pixel coordinates were first transformed to the 3D Cartesian reference frame of the Kinect camera with the help of the measured depth data and then transformed to the radar reference frame. The positions of the xiphoid and navel were estimated directly from the shoulder and hip positions.

To train the model, a dedicated loss function was used, defined as the mean absolute value of the angle between the lines connecting the radar to the true and predicted joint positions of the six considered points. The metric used was the mean absolute value of the distance between the true and predicted locations on the *x*–*z* plane in the Cartesian radar reference frame (with the *y*-axis corresponding to the direction and the azimuth and elevation both zero).

The algorithm used to classify the pose of the person as supine, prone, or lateral was very similar to that used to localize the skeleton joints. Instead of the final dense layer in the MLP, a softmax layer was included and the output of the model was one of the three classes. The categorical cross entropy was used as the loss function for training the model and the categorical accuracy was used as the evaluation metric.

All models were implemented and trained in Python with the TensorFlow library [41].

### 3.2. Movement Detection

The feasibility of detecting the movements of a person on the bed (in particular, pose changes and limb movements) based on MIMO radar data was studied by building a simple binary classifier (movement vs. no movement) for five-second measurement intervals.

The main idea behind the algorithm was to detect abrupt phase changes in the radar signals generated by relevant movements of the person’s body, under the assumption that these movements happened at a higher speed than the respiration-induced skin displacement. For each reduced radar data window, a movement indicator was first built by applying the following steps.

- Obtain the unwrapped phase signal $\zeta \left(t,\varphi_{k},\vartheta_{l}\right)=U\left\{\arg\left\{I\left(t,r_{j_{\mathrm{MA}}},\varphi_{k},\vartheta_{l}\right)\right\}\right\}$ at each azimuth–elevation combination with a mean reflection amplitude $\bar{A}\left[\varphi_{k},\vartheta_{l}\right]$ larger than a fixed threshold ${\bar{A}}_{\mathrm{TS}\;\mathrm{mov}}$.
- Calculate the phase changes between consecutive time steps $t_{i}$ and compute for each time step the number of azimuth–elevation pairs with a phase change larger than a fixed threshold $\Delta \zeta_{\min}$.
- Smooth the computed phase change values with a moving average filter over a time interval of 0.5 s.

The movement indicator was calculated for each five-second measurement interval and, if it exceeded a fixed threshold value $M_{\mathrm{TS}}$, the interval was classified as “with movement.”

### 3.3. Respiratory Movements

The respiratory movements were estimated during the time periods in which the measured person did not move and remained in the same position, which were identified based on the outputs of the movement detection algorithm. For each period, the corresponding 25 s windows of the reduced radar data $I\left[t,r_{j_{\mathrm{MA}}},\varphi_{k},\vartheta_{l}\right]$ were merged along the time axis, resulting in a complex array with dimensions $N_{\varphi }\times N_{\vartheta }\times N_{t}$, where $N_{t}$ is the number of time frames in the period. The estimated coordinates of the skeleton joints were then used to divide the person’s torso into chest and abdomen regions. For both regions, the respiratory motion was estimated independently based on the following steps:

- The unwrapped phase signal $\zeta \left(t,\varphi_{k},\vartheta_{l}\right)=U\left\{\arg\left\{I\left(t,r_{j_{\mathrm{MA}}},\varphi_{k},\vartheta_{l}\right)\right\}\right\}$ at each azimuth–elevation combination in the considered region was filtered in the band pass frequency range of 0.05 Hz to 6 Hz, s.t. the first harmonics of the breathing movement were included.
- The weighted average of the filtered signals was calculated, with the mean reflection amplitude of each point as the weight.

To estimate the breathing rate, a short-time Fourier transform (STFT) with a window length of 40 s was applied to the signal of the abdomen region. The breathing frequency at a given time step was then calculated by finding the maximum of the summed power of the fundamental frequency and the corresponding harmonics.

An estimate of the phase shift between the respiratory motion of the chest and the abdomen was computed by considering a 15 s window of the extracted respiratory movements of the abdomen $r_{a}^{t}$ and the chest $r_{c}^{t}$, centered around the considered time *t*, and by calculating the angle

(12) $$\begin{array}{c} \Theta \left(t\right)=\cos^{-1}\left(\frac{\langle r_{a}^{t},r_{c}^{t}\rangle }{∥r_{a}^{t}∥∥r_{c}^{t}∥}\right) \end{array}$$

between the two signals, where 〈·,·〉 and ∥·∥ denote the standard scalar product and the Euclidean norm, respectively. If the signals $r_{a}^{t}$ and $r_{c}^{t}$ were sinusoids of the same frequency with sufficient duration, then (12) was a good estimate of their phase difference. The computed value could be used to detect paradoxical breathing episodes, as these were often characterized by a significant phase shift between the respiratory motion of the chest and the abdomen [30].

## 4. Measurements

In this section, the measurement setup is first explained in detail and then the conducted measurement campaign is described. The results obtained through the application of the algorithms defined in Section 3 to the data collected during the measurement campaign are then presented. Finally, the results and possible improvements are discussed.

### 4.1. Measurement Setup

**Figure 5:**
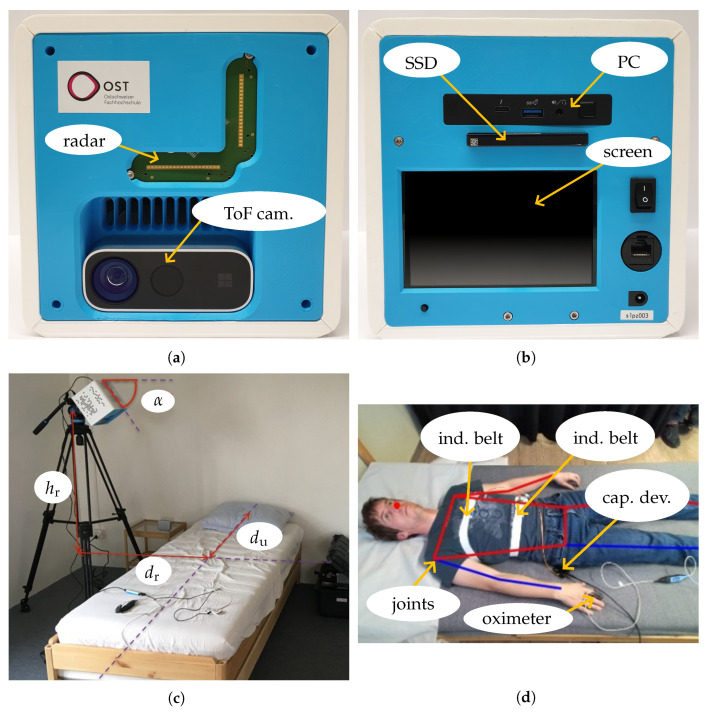
Measurement system in (**a**) front view and (**b**) rear view, (**c**) setup geometry next to the bed, and (**d**) reference systems for respiration and PPG signal with capturing device.

The measurement system consisted of a Mini-Circuits IMAGEVK-74 MIMO radar based on a Vayyar radio-frequency integrated circuit (RFIC) and a Microsoft Kinect Azure DK ToF camera mounted in a dedicated 3D-printed housing (see Figure 5a). The MIMO radar with an L-shaped array of 20 TX and 20 RX antennas operated in the 60 GHz band with a very high usable RF bandwidth of up to 7 GHz. Employing an SFCW modulation format, each TX sent a sequence of discrete mono-frequent signals in TDM mode. This sequence was repeated at a rate of 20 Hz to obtain information on the motion of the person and different parts of their body and their vital parameters. To reduce the level of sidelobes, the calibration method in [42] was used prior to the measurements. The Kinect camera was integrated as a reference system to track the position of the measured person on the bed based on the captured 1 Mpx depth and 2 Mpx RGB images recorded every 0.5 s. The locations of the joints were determined from the RGB image by using Google’s Mediapipe Pose algorithm. The data used for the evaluations presented in Section 4.3 were inspected by visualizing the RGB images with the estimated joints positions and manually correcting the wrong ones. The uncertainty in the obtained joints locations was estimated to be smaller than 5 cm. This information was combined with the depth image to estimate the joint locations in 3D coordinates. All radar and camera settings and derived parameters used are summarized in Table 1.

An Intel NUC 11 type NUC11PAHi7 PC was used to allow standalone operation, as depicted in Figure 5b. Measurement configuration and control could be conducted with a user interface on the integrated touchscreen, and data were saved by a background service to a pluggable WD Blue 3D NAND SSD with a capacity of 4 TB. For the training of the algorithms, additional attributes of the test subjects (such as height, weight, and gender) could be defined via a user interface and stored in a file containing personal data. Windows 10 Pro was used as the operating system. As it was not possible to perform real-time operations, UTC timestamps with a precision of 1 ms were added to the measurement data of all devices. For instance, individual radar images sampled at a default interval of 50 ms were recorded with a timestamp that showed the actual sampling time. Two dedicated services captured the radar and depth-camera data streams. As soon as a new dataset became available, the services appended the current system timestamp. This approach inevitably introduced a brief latency between the physical acquisition and its registration; however, the delay was negligible, because the Kinect’s inherently low sampling rate rendered small temporal offsets practically inconsequential. The rate at which data were recorded, when applying the settings in Table 1, was approximately 100 GB/h. One-third of the data were for RGB and depth images and two-thirds were for the radar ADC samples. This measurement system was primarily designed for apnea overnight screening and did not require real-time evaluation. It should be mentioned that the parameter estimation described in Section 3 could be implemented as an additional background process operating on sequences with a duration of 25 s to avoid storing a large volume of data. Furthermore, significant compression of radar data was possible by preprocessing raw ADC values to 2D radar images prior to storing them.

The ideal location for the measurement system would be directly above the person, especially above the torso. For home use, such a placement of the device is difficult as this requires equipment, such as a camera crane as device holder. Another suitable location would be close to the head, for instance, on a bedside table. To observe the respiration of a person, this setup should not be chosen due to the poor visibility of the chest and abdomen region as electromagnetic waves are primarily reflected away from the radar. Thus, the measurement system was placed next to the bed (see Figure 5c). The parameters were chosen as follows: α= 55°, the distance to the center of the bed $d_{r}=$ 80 cm, and $h_{r}=$ 90 cm with respect to the surface of the bed to see the person completely in the ±60° FoV. Since the bed had a length of 2 m, $d_{u}=$ 90 cm ensured that the upper part of the body was captured well. The main direction of radiation was toward the center of the bed. Putting the measurement system in an elevated position avoided strong undesired reflections from the bed frame.

As reference systems (shown in Figure 5d) for tracking the respiration-induced torso movements separately at the chest and abdomen, two disposable type 9007 inductive belts manufactured by SleepSense were used. The resonant frequency of both channels was measured by a Texas Instruments LDC1612 inductance-to-digital converter at 200 Hz in a time-multiplexed manner, and data were transferred to a capturing device via an I2C interface. The capturing device contained an STMicroelectronics STM32G0 microcontroller to perform the processing of raw respiration data and forwarding to the Intel NUC via a universal asynchronous receiver transmitter (UART) to the USB bridge. The photoplethysmogram and blood oxygen saturation were recorded with a Nonin Xpod sensor to track the measured heart rate and heart rate variability. The raw PPG signal was processed directly by the Nonin Xpod and the relevant values were derived autonomously. The communication with the capturing device was based on the standard commands for programmable instruments (SCPI) protocol.

### 4.2. Measurement Campaign

The described measurement setup was used to collect data in a measurement campaign with 23 volunteers. All procedures performed in studies involving human participants were in accordance with the ethical standards of the national research committee and the 1964 Helsinki declaration and its later amendments or comparable ethical standards. This study was exempt from approval by Cantonal Ethics Committee Zurich (no. 2022-01305) due to the early stage of the technology and low risk due to the contactless nature of the device. There was no need for an approval as this study did not fall into any definitions of the Swiss Federal Human Research Act. A summary of the characteristics of these volunteers is given in Table 2. To save time and induce larger variations in poses and vital parameter values, the volunteers were awake and were asked to follow predefined measurement protocols during two measurements, which had a duration of about one hour each.

**Figure 6:**
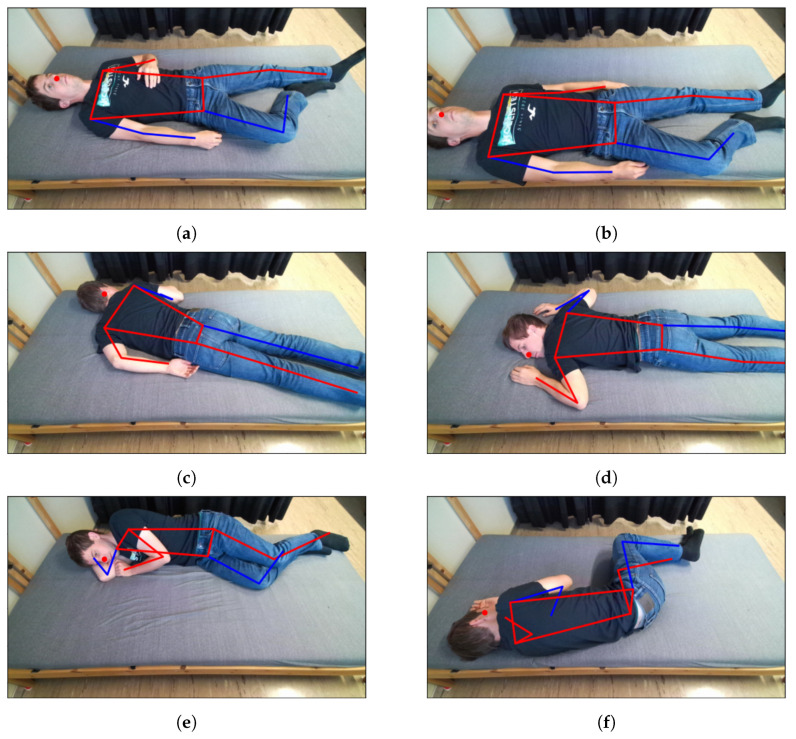
RGB images of example poses (**a**–**f**) that were measured during the measurement campaign. The skeleton coordinates plotted on the images are the ones obtained from the Google Mediapipe Pose algorithm.

The first measurement consisted of changing the body position on the bed approximately every minute while lying in a relaxed position, breathing naturally, and not moving between the pose changes. For each person, 45 different poses were measured: 15 for each class (supine, prone, and lateral), of which three were with the person covered by a blanket. The poses differed by the positioning of the person on the bed surface, with some performed at the center of the bed and others closer to the four edges of the bed (top, bottom, and lateral), and by the locations of arms and legs. Some illustrative examples of the poses measured for one person are given in Figure 6. This first measurement was performed without reference systems (chest belts and PPG) and the resulting data were used only to train and test the pose estimation algorithms.

The second measurement was similar to the first one, with longer intervals (two minutes) between pose changes. For each person, 18 different poses were measured: six for each class (supine, prone, and lateral), of which three were with the person covered by a blanket. This measurement was performed with inductive belts and a PPG device, and the data were used for both the pose and vital parameter estimation algorithms.

Additional measurements were performed for eight individuals to simulate episodes with asynchronous chest–abdomen respiratory movement with a duration of about 10 s while lying at the center of the bed in a supine, prone, or lateral position. The episodes were simulated by holding the breath and shifting the air in the lungs from the abdomen to the chest region and back at a rate similar to the normal respiratory rate. Some additional measurements were also performed with 10 persons regularly executing movements of single limbs (an arm or leg) with an amplitude varying between 5 cm and 30 cm and a duration of 1 s to 5 s. For each measurement, the person performed 41 single limb movements and four pose changes.

### 4.3. Results

After applying the preprocessing steps described in Section 2.2 to each data window of 25 s, a reduced dataset with 500 discrete time steps and $N_{r}=72$ points in the distance dimension, $N_{\varphi }=17$ points in the azimuthal dimension, and $N_{\vartheta }=25$ points in the elevation dimension was obtained. An example of the resulting azimuth–elevation array for the second phase evolution indicator $\alpha_{2}$ during one measurement with a person in supine position is shown in Figure 3.

Recognizing the presence of a person lying on the bed has proved to be feasible with high accuracy. The simple binary classifier based on the values of the first phase indicator $\alpha_{1}$ was tested on the data from the measurement campaign and on two additional short measurements without a person on the bed, achieving a classification accuracy of 100%.

For the pose classification algorithm and the regression algorithm for the coordinates of the key body joints, data from 20 people were used as the training set and the data of three other people were used as the test set. Each data point corresponded to a data window of τ=25s, during which the measured person was lying relaxed on the bed in a specific position, breathing naturally and not moving. A six-fold cross-validation procedure was used for hyperparameter tuning with the Optuna framework [43]. It is important to note that the data from a single person were not split over multiple folds.

**Figure 7:**
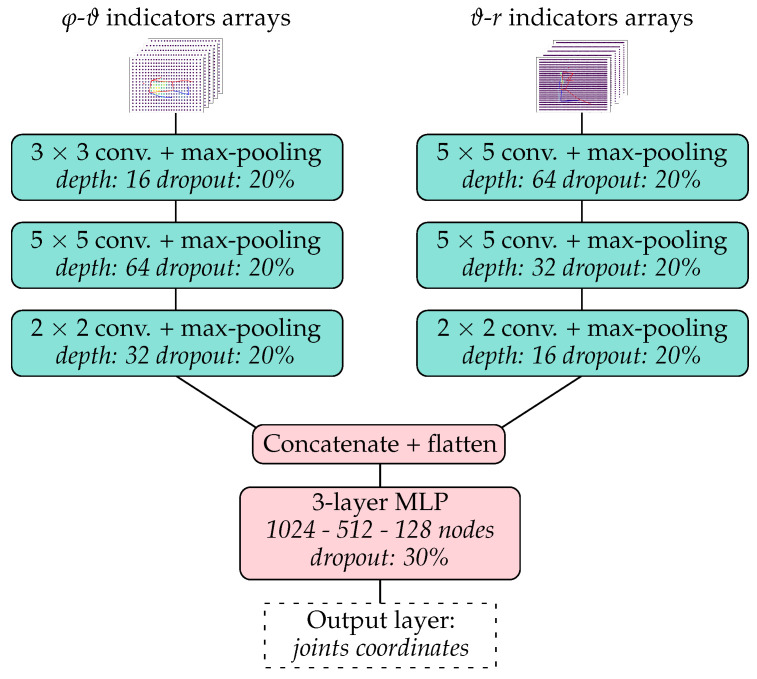
Schematic representation of the best model architecture for joint coordinates regression obtained via hyperparameter tuning. The size of the max-pooling layers was 2 × 2.

**Figure 8:**
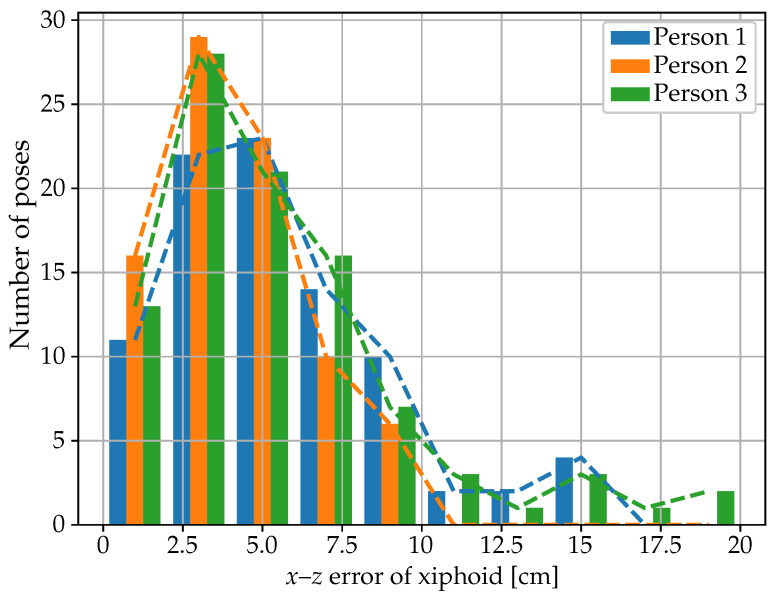
Frequency distribution of the localization error on the *x*–*z* plane of the xiphoid for the three people in the test set.

**Figure 9:**
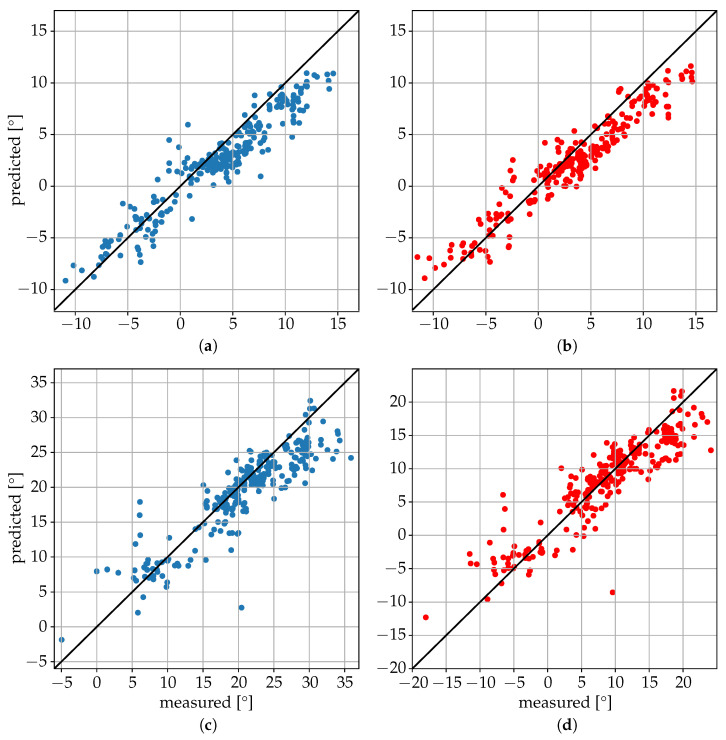
Estimation of azimuth angle for xiphoid (**a**) and navel (**b**) and elevation angle for xiphoid (**c**) and navel (**d**) for the three people in the test set.

The main hyperparameters of the best model architecture for the joint coordinates regression algorithm, obtained after hyperparameter tuning with Optuna with 200 trials, are illustrated in Figure 7. The results obtained on the test dataset are summarized in Table 3 and Table 4 and Figure 8 and Figure 9. The mean absolute error (MAE) for the xiphoid and navel locations on the radar *x*–*z* plane was smaller than 5 cm, and more than 90% of the measured poses had an error smaller than 10 cm (Figure 8). As a baseline comparison, a model that estimated the mean value of the training dataset was evaluated on the test dataset and reached an MAE in the localization of the xiphoid of about 13 cm. The procedure was repeated with different choices of the three individuals used for the test dataset and similar results were obtained, with the mean absolute error in the localization of the xiphoid and navel varying between 3.5 cm and 8 cm depending on the person. For the three people in the test set, the azimuth and elevation angles are shown in Figure 9 in the upper and lower plots, respectively. The value on the vertical axis corresponds to the estimated angle and the value on the horizontal axis corresponds to the measured reference angle. A total of 288 poses is shown in the form of individual dots.

It was interesting to observe the variability in the results between different individuals in the test dataset, which were likely caused by their different shapes and characteristics and their representation in the training dataset. The algorithm seemed to have more difficulty in localizing the joints in the lateral position, with the smallest errors obtained in the supine position (see Table 4).

Hyperparameter tuning with Optuna was also applied to the pose classification algorithm, with the best architecture achieving a categorical accuracy on the test data of about 93% (see Table 5). The procedure was repeated with different choices of the three individuals used for the test set and similar results were obtained, with the categorical accuracy varying between 90% and 95% depending on the choice of subjects.

The simple algorithm for movement detection described in Section 3.2 was applied to the limb movement measurements with an amplitude threshold ${\bar{A}}_{TS\;\mathrm{mov}}=30\;\mathrm{dB}$, a threshold for phase change $\Delta \zeta_{\min}=1.5\;\mathrm{rad}$, and a threshold for the movement indicator $M_{\mathrm{TS}}=5$. The resulting confusion matrix of the binary classification of five-second time intervals for the 10 measurements is given in Table 6. Although simple, the developed procedure with a categorical accuracy of 98.6% showed good classification performance. Only some small arm movements and small leg movements (displacement of about 5 cm) were not detected correctly. These movements did not generate relevant changes in the electromagnetic signals reflected to the radar and, thus, it was not possible to recognize them with the described approach.

**Figure 10:**
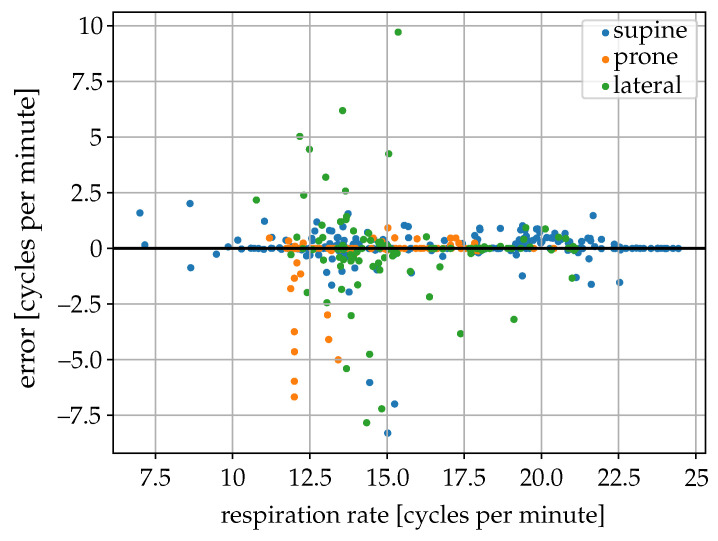
Scatter plot of the error of the radar-based respiration rate estimate from the abdomen region versus the respiration rate estimated from the chest belt signal (reference).

The estimated breathing rate from the radar data was compared with the estimate from the inductive belt signals for 512 different 2 min sequences without movements of seven different individuals in different poses (264 supine, 126 prone, 126 lateral) (Figure 10). The breathing rate measured with the inductive belts among the 2-min sequences used varied between 7 and 24 cycles per minute. In the supine position, an MAE of the estimated breathing rate of about 0.33 cycles per minute and a 90-percentile of the absolute error of 0.88 cycles per minute were obtained. In the prone position, the obtained MAE was 0.06 cycles per minute and the 90-percentile of the absolute error was 0.25 cycles per minute, whereas, in the lateral position these values were respectively 0.80 and 2.80 cycles per minute. Most of the sequences with errors larger than two cycles per minute, mainly in the lateral position and representing only about 3% of the sequences analyzed, were related to the low quality of the radar signal or the presence of disturbances (probably caused by very small body movements).

**Figure 11:**
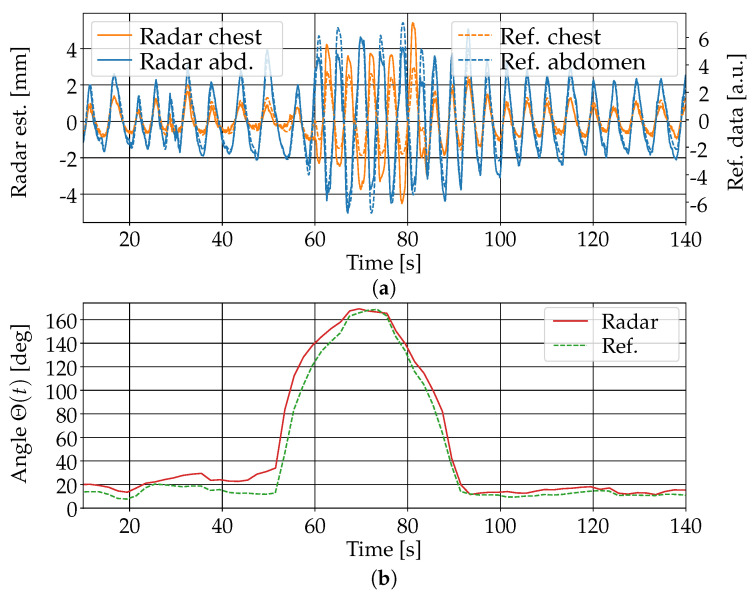
Simulated asynchronous chest–abdomen respiratory movement starting after 60 s with a person in supine position. (**a**) Comparison of the breathing movements at the chest (orange) and abdomen (blue) based on radar data (continuous line) and on inductive belts (dashed line). (**b**) Phase difference between the respiratory movement at the chest and abdomen estimated from the radar data (green) and inductive belts (red).

The estimation of separate respiratory movements of the chest and abdomen using the algorithm in Section 3.3 was in principle feasible. Figure 11 shows the results of the estimation for one person in the supine position. The person was covered with a blanket and simulated asynchronous chest–abdomen respiratory movement for about 10 s in the middle of the measurement sequence. The comparison with the signals obtained from the inductive belts showed good agreement both in the breathing movements of the chest and abdomen and the values of the indicator for their phase difference. Note that the amplitude of the movement estimation from the radar data could not be directly compared to the one obtained from the belts because of the different measurement principles used by the two systems. The signals were appropriately scaled to enable a direct visual comparison. The same evaluation was performed with similar measurements of eight different individuals in three different poses (supine, lateral, and prone). In the supine position, a good agreement between the estimated phase difference and the reference value computed from the chest belt signals (difference smaller than 30 degrees) was observed for all eight persons. In the prone position, similar results were obtained for six persons, while, for two persons, the phase difference error reached about 80 degrees due to greater noise in the reflection signals from one of the two torso regions. In the lateral position with the person facing the radar device, good results were obtained for all eight persons, with a maximum phase difference error of about 50 degrees. In the lateral position with the person facing away from the radar, the computed signals were much more noisy and the asynchronous respiratory movement was only in some cases barely recognizable in the data.

### 4.4. Discussion

The obtained results show that, in principle, it is feasible to use mm-wave MIMO radar data to recognize the presence of a person sleeping on a bed, classify their sleeping pose (supine, prone, or lateral), and measure the location of their torso (in particular, the chest and abdomen) with good accuracy. We expect in particular that with a larger dataset (more than 50 people with variable body shapes) it should be possible to achieve a categorical accuracy in pose classification greater than 95% and an MAE on the *x*–*z* plane in the localization of the xiphoid and navel below 5 cm in all sleeping positions. In the lateral position, the obtained accuracy was lower than in the prone or supine position, which was probably caused by several factors. In the lateral position facing the radar device, people often positioned their arms in front of the torso, thus disturbing the signal reflection. In this position, the torso also has a cross section lower than the radar device, and the respiration movements are typically smaller, as the torso expansion during respiration takes place both in the chest–abdomen and back directions.

It is, however, important to note that due to the specular nature of microwave reflection on the skin, the measured signals were strongly direction-dependent, so that the algorithms performed well only if the measurement system was placed in approximately the same position with respect to the bed as during the collection of the training data. A shift in the position of the system of 10 cm was enough to cause a significant reduction in the accuracy of the models. This strong device position dependence could be reduced by building more robust algorithms through a slight variation of the position of the measurement device around a defined reference point during the collection of the training data.

The results also show that as the influence of blankets on the measured mm-wave radar data was very small, it is possible to collect most of the training data without cover and obtain similar accuracy on the test data measured with and without a blanket, thus significantly reducing the data collection effort.

The detection of body movements is also possible in principle. Only the detection of small limb movements (<5 cm) seemed to be especially difficult, depending on the position and orientation of the limb with respect to the radar. Based on the data collected, we expect that developing an algorithm for the classification of movements (e.g., pose changes, arm movements, leg movements) should also be possible with good accuracy. This information could then be used to characterize persons movement during sleep in overnight measurements.

Separate tracking of the respiratory movement at the chest and abdomen proved to be feasible, with good accuracy in supine, prone, and lateral positions with the person facing the radar device. Depending on the orientation and shape of the measured person, it may be that this separate tracking is not always possible or has low accuracy as, due to the specular nature of the radar signals, no relevant reflections are available from one of the two regions of the torso. For the setting used in the described analysis, the lateral sleeping position with the person facing away from the radar device turned out to be particularly unfavorable for measuring separate respiratory movements of the chest and abdomen.

By using a MIMO radar with higher output power, further studies could be conducted to measure heart rate and the inter-beat intervals of the pulse, comparable to [44]. Another possibility is measuring the blood pressure in the region of the sternum [45]. By using position information in combination with beam-forming, the necessity of applying antennas to the body surface could be overcome.

## 5. Conclusions and Outlook

In this work, we proposed a MIMO radar system concept that uses a forked CNN architecture for pose estimation, trained with data obtained by a ToF camera. An MAE for the localization of about 5 cm in the *x*–*z* plane and a pose classification categorical accuracy between 90% and 95% were achieved. In contrast to commonly used CW radar, the presented prototype allows for the recognition of different parts of the body and limb movements and the separate recording of respiratory movements in the chest and abdomen. Events of paradoxical breathing could be detected with the developed system by tracking and evaluating the phase shift between chest and abdomen respiratory movement.

Further improvements of the measurement system are possible by choosing a radar system with higher output power to enhance the SNR. This would ensure that heart rate and HRV could be measured during the night as additional health parameters. A larger measurement campaign to collect more data for training the models would also be beneficial to obtain better localization and pose classification results, as well as increase the robustness and generalizability of the models over different body shapes. Finally, overnight measurements with sleep apnea patients and additional reference systems would be required to fully evaluate the system.

## Figures and Tables

**Table 1:** Radar and camera system parameters.

Symbol	Parameter	Value
$f_{0}$	RF center frequency	65.5 GHz
*B*	RF bandwidth	5.2 GHz
*L*	frequency steps	75
Δr	range resolution	5.75 cm
$r_{\max}$	maximum range	2.1 m
$\Delta t_{\mathrm{radar}}$	radar measurement repetition time	50 ms
$v_{\max}$	maximum velocity	22.9 mm/s
*M*	number of RX antennas	20
*N*	number of TX antennas	20
$d_{\mathrm{rx}}$, $d_{\mathrm{tx}}$,	inter-element spacing	≈2.1 mm
Δφ, Δϑ	maximum angular resolution	≈13°
τ	time interval for data processing	25 s
$N_{r}$	FFT size range	128
$N_{\varphi }$	FFT size azimuth	64
$N_{\vartheta }$	FFT size elevation	64
$\Delta t_{\mathrm{camera}}$	camera measurement repetition rate	500 ms
#${\mathrm{px}}_{\mathrm{RGB}}$	camera RGB resolution	2 Mpx
#${\mathrm{px}}_{\mathrm{depth}}$	camera depth resolution	1 Mpx
$\varphi_{\max}$, $\vartheta_{\max}$	radar and camera angular FoV	≈±60°

**Table 2:** Summary of the characteristics of the volunteers in the measurement campaign.

Parameter	Male	Female
Number	10	13
Age	23 to 37	23 to 48
Weight [kg]	54 to 93	50 to 95
Height [cm]	165 to 187	155 to 178
BMI	18.7 to 27.8	18.6 to 32.9

**Table 3:** Mean absolute error in the localization of the skeleton joints on the radar *x*–*z* plane of the test data.

Point	MAE Test Data [cm]	MAE Person 1 [cm]	MAE Person 2 [cm]	MAE Person 3 [cm]
xiphoid	4.6	4.7	3.5	5.3
navel	4.5	4.6	3.5	5.2
left shoulder	6.3	6.5	5.7	6.6
right shoulder	6.1	5.3	6.1	6.8
left hip	5.5	5.2	5.2	6.1
right hip	5.5	5.0	4.8	6.4

The error values for each of the three persons included in the test set are listed separately.

**Table 4:** Mean absolute error in the localization of the skeleton joints on the radar *x*–*z* plane of the test data.

Point	MAE Test Data [cm]	MAE Supine [cm]	MAE Lateral [cm]	MAE Prone [cm]
xiphoid	4.6	3.5	5.6	4.5
navel	4.5	3.8	5.4	4.2
left shoulder	6.3	4.8	7.5	6.5
right shoulder	6.1	5.1	7.3	5.9
left hip	5.5	5.4	6.1	5.0
right hip	5.5	4.5	7.1	4.7

The error values for each pose class are listed separately.

**Table 5:** Confusion matrix of the pose classification algorithm on the test set.

		**pred.**		
		lateral	prone	supine
**meas.**	lateral	89	7	0
	prone	1	94	1
	supine	5	5	86

**Table 6:** Confusion matrix of the movement detection algorithm applied to 10 limb movement measurements.

		**pred.**	
		movement	no movement
**meas.**	movement	391	20
	no movement	5	1423

## Data Availability

The data presented in this study are available on request from the corresponding author. The data includes optical references that can be used to identify volunteer participants in the studies.
